# Magnetotelluric Signal-Noise Separation Using IE-LZC and MP

**DOI:** 10.3390/e21121190

**Published:** 2019-12-04

**Authors:** Xian Zhang, Diquan Li, Jin Li, Yong Li, Jialin Wang, Shanshan Liu, Zhimin Xu

**Affiliations:** 1 Key Laboratory of Metallogenic Prediction of Nonferrous Metals and Geological Environment, Monitoring Ministry of Education, School of Geoscience and Info-Physics, Central South University, Changsha 410083, China; zhangxian128@csu.edu.cn; 2 Hunan Provincial Key Laboratory of Intelligent Computing and Language Information Processing, Hunan Normal University, Changsha 410081, China; 201970293227@smail.hunnu.edu.cn (J.W.); 201970293224@smail.hunnu.edu.cn (S.L.); 3 Key Laboratory of Geophysical Electromagnetic Probing Technologies of Ministry of Natural Resources, Institute of Geophysical and Geochemical Exploration, Chinese Academy of Geological Science, Langfang 065000, China; liyong@igge.cn; 4 Hunan Key Laboratory of Nonferrous Resources and Geological Hazard Exploration, Changsha 410083, China; 5 Hebei Instrument & Meter Engineering Technology Research Center, Chengde Petroleum College, Chengde 067000, China; xuzhimindx@126.com

**Keywords:** magnetotelluric (MT), signal-noise separation, information entropy (IE), Lempel–Ziv complexity (LZC), matching pursuit (MP)

## Abstract

Eliminating noise signals of the magnetotelluric (MT) method is bound to improve the quality of MT data. However, existing de-noising methods are designed for use in whole MT data sets, causing the loss of low-frequency information and severe mutation of the apparent resistivity-phase curve in low-frequency bands. In this paper, we used information entropy (IE), the Lempel–Ziv complexity (LZC), and matching pursuit (MP) to distinguish and suppress MT noise signals. Firstly, we extracted IE and LZC characteristic parameters from each segment of the MT signal in the time-series. Then, the characteristic parameters were input into the FCM clustering to automatically distinguish between the signal and noise. Next, the MP de-noising algorithm was used independently to eliminate MT signal segments that were identified as interference. Finally, the identified useful signal segments were combined with the denoised data segments to reconstruct the signal. The proposed method was validated through clustering analysis based on the signal samples collected at the Qinghai test site and the measured sites, where the results were compared to those obtained using the remote reference method and independent use of the MP method. The findings show that strong interference is purposefully removed, and the apparent resistivity-phase curve is continuous and stable. Moreover, the processed data can accurately reflect the geoelectrical information and improve the level of geological interpretation.

## 1. Introduction

The magnetotelluric (MT) method, an electromagnetic exploration method proposed in the early 1950s [1,2], measures the orthogonal electric-magnetic fields at the Earth’s surface to obtain the distribution of the underground geoelectric structure. The MT method has a series of characteristics such as its utilization of the natural field source, its large exploration depth, simple-formed plane wave theoretical impedance, and simple geological interpretation [3,4,5,6]. Whether for research into deep geological structures or the exploration of deep concealed blind ore bodies, the MT sounding method is an effective means. However, the MT signal itself is extremely weak and random, making it impossible to avoid complicated electromagnetic interference environments, leading to data contamination from noise and low data collection reliability. Thus, useful MT signals can be extracted from the corrupted data, and strong interference is purposefully suppressed, which is essential for a reliable geophysical data interpretation.

For nonlinear and non-stationary MT signals, scholars have proposed a large number of MT signal processing methods, such as the remote reference method [7], robust impedance estimation [8,9], wavelet transform [10], Hilbert–Huang transform [11], mathematical morphology filtering [12], variational mode decomposition [13], among others. All the above methods can filter the entire data set and improve the quality of MT data to some extent. However, some useful MT signals are also filtered out due to the lack of identification. The emerging developments in MT signal-noise identification over recent years have presented a new processing mode for MT signal-noise separation [14,15]. These methods distinguish whether the signal is contaminated by noise based on several characteristic parameters extracted from the signal. Although these methods have produced good results, it still takes a long time to extract the features during data processing.

Thus, the main focus of this paper is still the analysis and application of the complexity index. Shannon first used the concept of entropy to evaluate the randomness information theory (IE); where entropy is a simple and efficient feature that characterizes the complexity of a system [16]. Low entropy of an MT time series indicates low complexity, whereas a higher value of entropy would imply higher complexity, that is, low entropy corresponds to an MT signal with interference, otherwise it is a useful MT signal. Kolmogorov proposed the significant concepts of system complexity in 1965s [17]. Given the incalculability of the Kolomogorov complexity, the Lempel–Ziv complexity (LZC) algorithm was designed to describe the complexity of the signal. Since the size of the value reflects the degree of chaos, the LZC is also applied to evaluate the nonlinear dynamics of the MT time series. Nowadays, IE and LZC are widely used as measures of dynamical complexity in several applications [18,19,20].

In this paper, a novel method was proposed for MT signal-noise separation using IE-LZC and matching pursuit (MP). The IE-LZC characteristics were developed to analyze the essential features of MT in combination with the fuzzy c-means (FCM) clustering algorithm for MT signal-noise identification or with the selective use of matching pursuit (MP) as the de-noising algorithm. The IE and LZC were extracted from each segment of the MT signal as characteristic parameters. The FCM clustering is a partition-based clustering algorithm [21] designed to identify the signal and noise automatically. The idea is to maximize the similarity between objects that are divided into the same cluster, and the similarity between different clusters is the smallest. The MP algorithm is a highly adaptive time-frequency signal decomposition and approximation method that is commonly used to suppress signals identified as strong interference. We applied the proposed method for the clustering analysis of signal samples collected from the Qinghai test site and the measured sites; where the results were compared to those obtained using the remote reference method and independent use of the MP method. According to the findings, the denoised data from the proposed method closely resembled the original undisturbed data in terms of the essential characteristics, and the geoelectric structure information of the measured site was accurately reflected in the results.

## 2. Methods and Materials

Based on the essential characteristics of the MT signal and noise, the collected MT data is often due to factors such as topographical structure and the human electromagnetic environment, and various electromagnetic noises inevitably interfere with it. However, the complex electromagnetic interference sources cause some disturbances to show robust features in the time-series and frequency spectrum, while other useful signals do not show any features in the time-series and frequency spectrum [22]. Therefore, the proposed method was processed for time-series and it was composed of three steps: characteristic extraction (IE and LZC), clustering analysis (FCM), and the de-noising algorithm (MP). From the above steps, IE-LZC and MP are presented, respectively. 

### 2.1. Information Entropy (IE)

Information entropy is a term describing the degree of uncertainty of the system [23,24], and therefore, it can be used to measure nonlinear and nonstationary MT signals. Lower entropy means less uncertainty of the information, that is, the MT signal with substantial interference in this paper. On the other hand, a useful MT signal contains more random and complex information. The information entropy is defined by Shannon as follows:

(1) $$H\left(X\right)=-\sum_{1}^{N}p\left(x_{i}\right)\log p\left(x_{i}\right),$$

where X is a discrete random variable with limited states, the probability of each state $X=x_{i},i=1,2,\ldots ,N$ is denoted as $p\left(x_{i}\right)=p\left(X=x_{i}\right)$.

### 2.2. Lempel–Ziv Complexity (LZC)

The Lempel–Ziv complexity is a nonlinear method for coarse-graining the original signal to form a symbol series, and also for parsing data to estimate the complexity caused by the emergence of new sub-sequences within the symbol series [25,26].

Given $X=\left\{x_{1},x_{2},x_{3}\ldots \right\}$, denotes the original string sequence, among them, set a=mean(X(i)); if X(i)≥a, then x(i)=0, or else 1. Let S and Q be two subsequences of sequence X, and SQ is a concatenation of S and Q, while the sequence SQπ is derived from SQ after its last character is deleted (π implying deletion of the last character in the sequence) and v(SQπ) denotes the vocabulary of all different subsequences of SQπ.

(1) Initialization; complexity counter c(N)=1, $S{=x}_{1}$, $Q=x_{2}$, therefore, $SQ\pi =x_{1}$.
(2) Make $S=x_{1},x_{2},\ldots ,x_{r}$ and $Q{=x}_{r+1}$, then $SQ\pi =x_{1},x_{2},\ldots ,x_{r}$, if Q belongs to v(SQπ), then Q is a subsequence of SQπ, not a new sequence.
(3) Change Q to be $x_{r+1},x_{r+2}$ and judge whether Q belongs to v(SQπ). Repeat above step until Q does not belong to v(SQπ).
(4) If not, $Q=x_{r+1},x_{r+2},\ldots ,x_{r+i}$ is not a subsequence of $SQ\pi =x_{1},x_{2},\ldots ,x_{r+i-1}$, so increase c(N) by one.
(5) After that, $S=x_{1},x_{2},\ldots ,x_{r+i}$ and $Q=x_{r+i+1}$. Repeat the previous steps until Q is the last character. Therefore, the number of subsequences in X is c(N), which is the measure of complexity.

The complexity value is related to the sequence length N and c(N) must be normalized. If the number of different symbols is α, it has been shown that the upper bound of c(N) is as follows:

(2) $$c\left(N\right)<N/\left(\left(1-\varepsilon_{N}\right)\log_{\alpha }\left(N\right)\right)$$

where $\varepsilon_{N}$ is a small quantity and $\varepsilon_{N}\to 0\left(N\to \infty \right)$, and $N/\log_{\alpha }\left(N\right)$ is the upper limit of c(N),

(3) $$\underset{N\to \infty }{\lim}c\left(N\right)=b\left(N\right)=N/\log_{\alpha }\left(N\right)$$

For a binary conversion α=2, $b\left(N\right)=N/\log_{\alpha }\left(N\right)$. c(N) can be normalized by b(N) as:

(4) C(N)=c(N)/b(N)

C(N) is the normalized LZC. The larger the value of the LZC, the more complex the sequence is; that is, the MT signal has no interference.

In this paper, IE and LZC were used as robust characteristic parameters to distinguish MT signals from noise. Simultaneously, as the input of FCM clustering, it is used for signal-noise identification.

### 2.3. Matching Pursuit (MP)

The matching pursuit (MP) algorithm can be traced back to the overcomplete dictionary [27], that is, the signal is decomposed into a sparse representation coefficient on a non-orthogonal basis. The basic idea is to select the best atom to match the signal in each iteration and to constantly update the residual signal until iterative termination [28]. The MP algorithm is as follows:

Let dictionary $D={\left\{g_{\gamma }\right\}}_{\gamma \in \Gamma }$, where $g_{\gamma }$ refers to redundant atoms, and then, the signal f can be decomposed into:

(5) $$f=\langle f,g_{\gamma 0}\rangle g_{\gamma 0}+Rf$$

where $\langle f,g_{\gamma 0}\rangle g_{\gamma 0}$ is the projection of the signal on the atoms, Rf is the residual signal, and $g_{\gamma 0}$ is orthogonal to Rf, hence:

(6) $${\left\|f\right\|}^{2}={\left|\langle f,g_{\gamma 0}\rangle \right|}^{2}+{\left\|Rf\right\|}^{2}$$

To minimize the residual signal, we approximated the best atom chosen to maximize the inner product of the f and $g_{\gamma 0}$, and the residual signal. When iterating to n+1, the residual after approximating by n atoms can be expressed as:

(7) $$R^{n}f=\langle R^{n}f,g_{\gamma n}\rangle g_{\gamma n}+R^{n+1}f$$

Through N iterations, the signal f can be decomposed into:

(8) $$f=\sum_{n=0}^{N-1}\langle R^{n}f,g_{\gamma n}\rangle g_{\gamma n}+R^{n}f$$

Since the residual is exponentially decaying and thus ignored, the signal f can be approximated as:

(9) $$f=\sum_{n=0}^{N-1}\langle R^{n}f,g_{\gamma n}\rangle g_{\gamma n}$$

**Figure 1:**
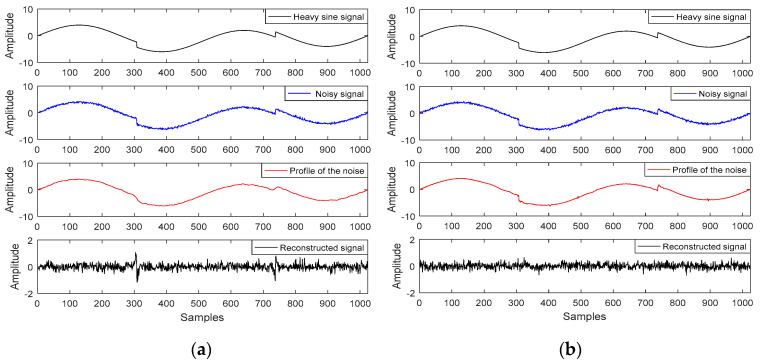
(Matching pursuit) MP de-noising effect of the ‘heavy sine’ signal under different atoms with (**a**) sin/dct atoms and (**b**) sym/db wavelet atoms.

Figure 1 shows the MP de-noising effect of the ‘heavy sine’ signal in MATLAB under different atoms, and the length of the ‘heavy sine’ signal was 1024. In the signal, we analyzed it by adding random noise. Among them, the dictionary in the MP algorithm only contains sine (sin) and discrete cosine (dct) atoms, as shown in Figure 1a, while Figure 1b displays the symlets (sym) and daubechies (db) wavelet atoms. It can be seen from Figure 1a that in the part of the signal mutation, the sin/dct atoms cannot accurately match the signal’s detail component, resulting in residual interference in the reconstructed signal. Therefore, the sym/db wavelet atoms can match the abrupt part of the ‘heavy sine’ signal.

To evaluate the MP de-noising performance of different atoms, the normalized cross-correlation (NCC), signal-to-noise ratio (SNR), and mean square error (MSE) were used for the quantitative analysis. Definitions of these parameters are as follows:

(1) NCC

(10) $$NCC=\frac{\sum_{i=1}^{n}f\left(i\right)g\left(i\right)}{\sqrt{\left(\sum_{i=1}^{n}f^{2}\left(i\right)\right)\left(\sum_{i=1}^{n}g^{2}\left(i\right)\right)}}$$

where f(i) and g(i) represent the ‘heavy sine’ signal and profile of the noise, respectively.

(2) SNR

(11) $$SNR=10\mathrm{lg}\frac{\sum_{i=1}^{N}f^{2}\left(i\right)}{\sum_{i=1}^{N}{\left[f\left(i\right)-g\left(i\right)\right]}^{2}}$$

(3) MSE

(12) $$MSE=\frac{1}{N}\sum_{i=1}^{N}{\left(f\left(i\right)-g\left(i\right)\right)}^{2}$$

Table 1 is the comparison between the de-noising performance of different atoms.

From Table 1 together with Figure 1, the sym/db wavelet atoms achieve a higher NCC and SNR, and a smaller MSE, which accurately matches the abrupt component signal, so that the reconstructed signal is more precisely preserved. To this end, the appropriate atoms were selected to match the stationary and abrupt signals effectively.

## 3. Experiments and Results

### 3.1. Step of the Proposed Method

The steps of the proposed method are detailed as follows:

1. Load the noisy MT data and equally divide it into the N segment of data.
2. Extract the IE and LZC of each segment of the MT data, and input to FCM clustering as signal-noise identification, automatically distinguishing the signal and noise.
3. The data segment identified as the MT signal is retained, and the MP technique eliminates the data segment identified as the MT noise, that is, the MP signal-noise separation.
4. The retained signal is combined with the denoised signal to reconstruct the useful MT signal.
5. Evaluate the apparent resistivity-phase curve and electromagnetic polarization direction.

### 3.2. Clustering Analysis of the Sample Library

In this research, a library of 200 data samples was used from field measurements [29]: where 50 samples were MT signals without interference collected from a remote area with no man-made activities in the Qinghai province. The rest of the contaminated data (referred to as “square wave interference, triangle wave interference, and pulse wave interference”) were collected from the ore concentration area.

The FCM clustering algorithm obtains the membership degree of each sample to all clustering centers by optimizing the objective function, thereby determining the category of the samples to achieve the purpose of automatic classification.

**Figure 2:**
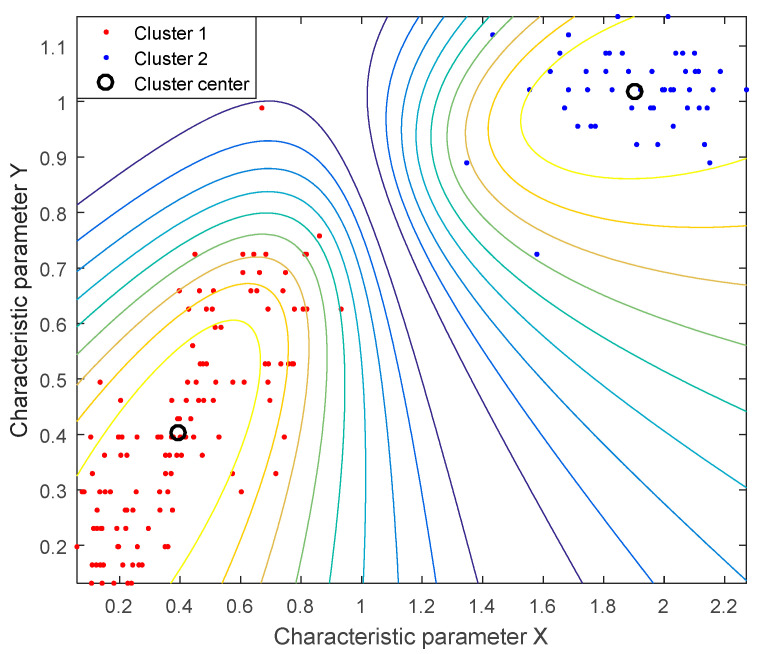
FCM clustering of sample library signals, where Cluster 1 represents MT signals with interference, and Cluster 2 represents MT signals without interference; Characteristic parameter X and Y are information entropy (IE) and the Lempel–Ziv complexity (LZC), respectively.

Figure 2 is the fuzzy c-means (FCM) clustering of the sample library signals; among them, cluster 1 represents the MT signals with interference and cluster 2 represents the MT signals without interference. Characteristic parameters X and Y are the information entropy (IE) and Lempel–Ziv complexity (LZC), respectively. Extract the IE and LZC characteristic parameters from the signals of the sample library and then input them to FCM clustering; calculate the Euclidean distance from the characteristic value to the cluster center, and then use contour lines to distinguish between the two types. Specifically, the contour lines indicate the shortest and longest distance from the samples to the cluster center in the same type, which can effectively divide the sample signals into different groups, thereby accurately identifying the MT signals without or with interference. Thus, IE and LZC are suitable for distinguishing MT data, and this sample library proved the feasibility of using FCM clustering for MT signal-noise identification.

### 3.3. Simulated Interference Analysis of the Qinghai Test Site Signal

The test site (QH401504) data was collected from remote areas untouched by human activities in the Qinghai province. The duration of data collection was about 19 h; where the pseudo-random sequence transmitted by the wide-area electromagnetic transmitter was implanted into the MT data collected in the first 1.5 h. During the remaining 17.5 h, the wide-field electromagnetic transmitter was turned off, which meant that the MT data was unaffected by the strong interference. To this end, we only analyzed the data collected during the last 17.5 h for this test site.

**Figure 3:**
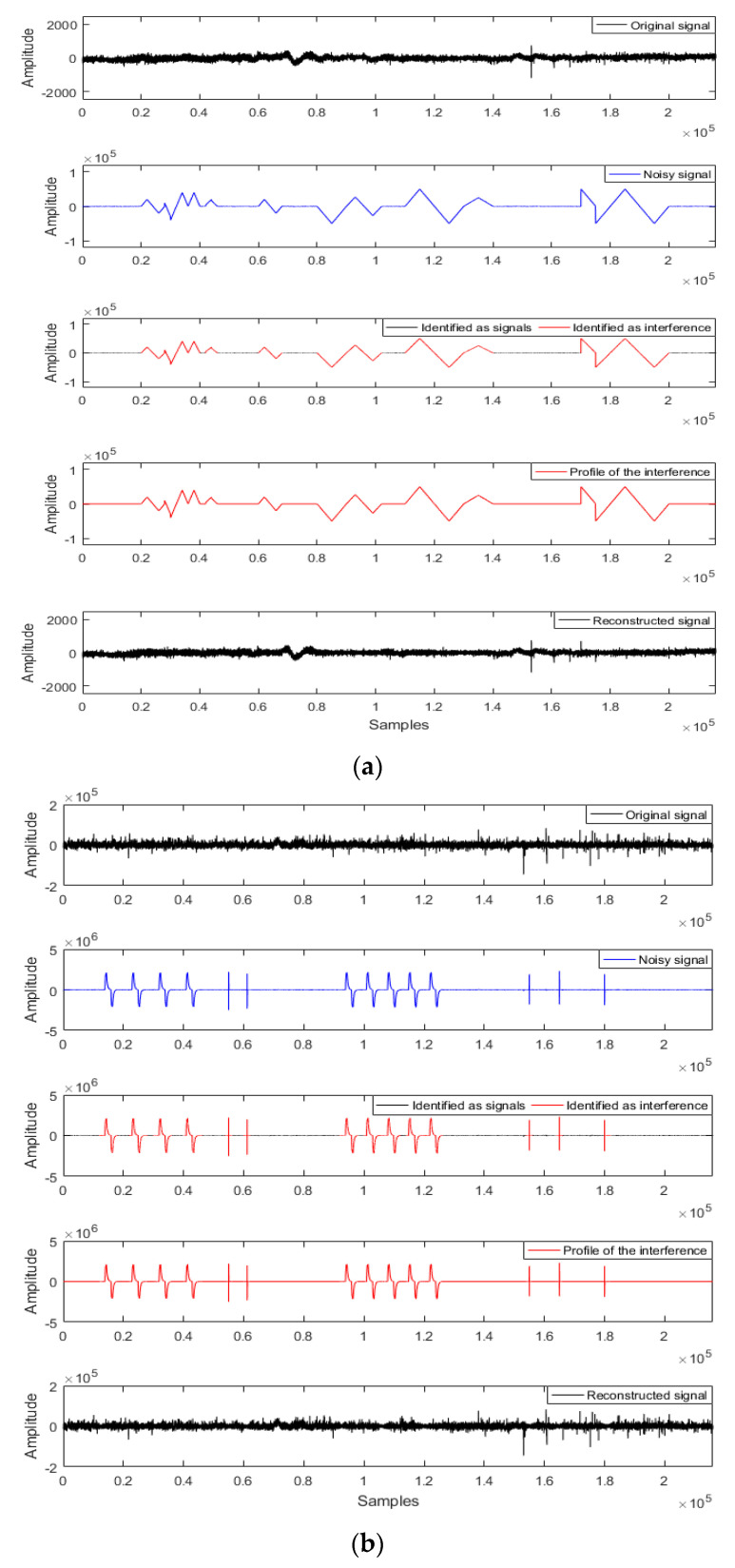

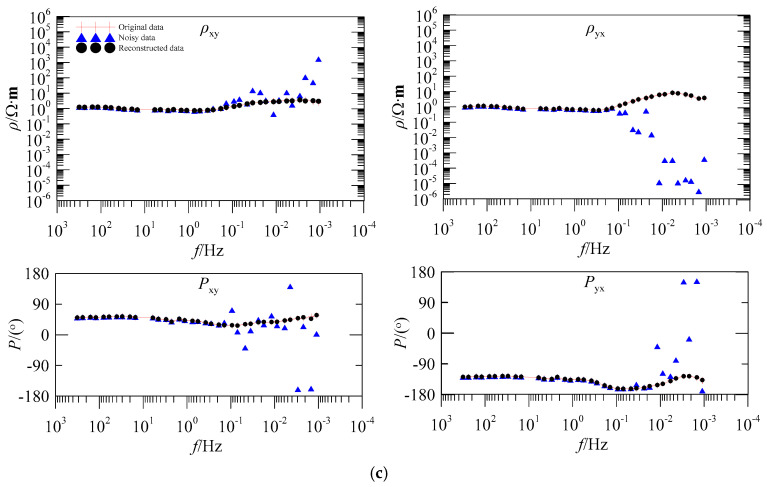
The Qinghai test site (QH401504) is disturbed by a noisy signal with (**a**) triangle wave interference of Ex and (**b**) charge and discharge triangle wave and pulse wave interference of Hx; (**c**) is a comparison of the apparent resistivity-phase curves.

Figure 3 shows that large-scale interference was added to the undisturbed data for signal-noise separation analysis and comparison of the denoised apparent resistivity-phase curves. The triangle wave interference, charge-discharge triangle wave interference, and pulse wave interference were added into the Ex or Hx channel, respectively. Figure 3a,b can effectively identify the artificially-added interference types by extracting the characteristic parameters and the FCM clustering analysis, thereby eliminating large-scale abnormal waveforms while retaining as many details of the original signal as possible for the reconstructed signal. Further comparison of the apparent resistivity-phase curves is shown in Figure 3c, where the apparent resistivity-phase curve of the original data is smooth and continuous. However, noisy data causes the apparent resistivity-phase curve to mutate or fall severely in the low frequency band. The apparent resistivity-phase curve of the reconstructed data using the proposed method was consistent with that of the original data. Therefore, the proposed method could suppress the MT abnormal waveform in a targeted manner and provided an effective way to analyze the subsequent measured data of the measured sites.

### 3.4. Signal-Noise Separation Analysis of the Measured Data

**Figure 4:**
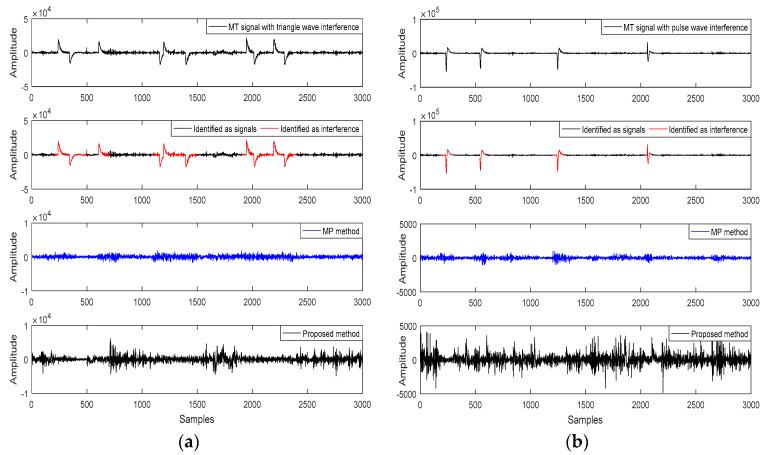
The signal-noise separation for the measured magnetotelluric (MT) data and comparison of using the MP method (**a**) triangle wave interference and (**b**) pulse wave interference.

To verify the effectiveness of the proposed method, Figure 4 shows the signal-noise separation of the measured data using the IE-LZC and MP technique. The triangle wave and pulse wave interference were identified and highlighted in red. The result confirmed that the proposed method could distinguish between signals and interference.

Large-scale strong interference was suppressed using the MP method for de-noising. Nevertheless, this method lacked signal-noise identification, which could lead to improper filtration of abundantly useful information making it hard to reserve non-interfered MT signals. In contrast, the proposed method not only quantitatively identified the signals and interference but it also suppressed the identified interference purposefully, thereby avoiding the over-processing of the MP method and abundantly preserving the slow-change components at low frequencies.

### 3.5. Apparent Resistivity-Phase Curve of the Measured Sites Analysis

**Figure 5:**
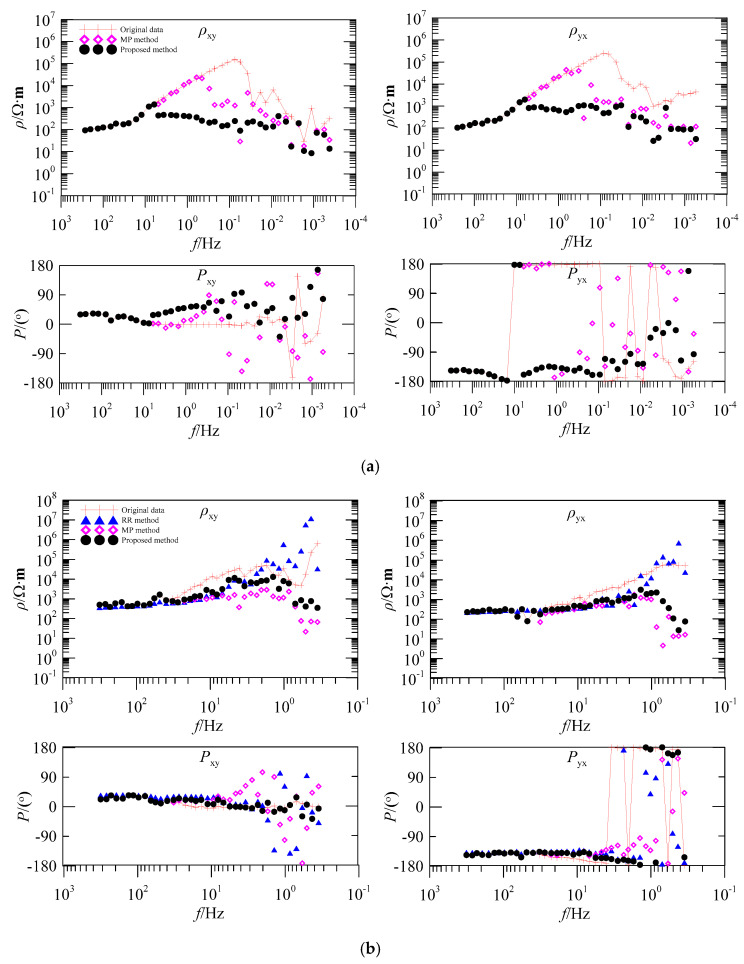

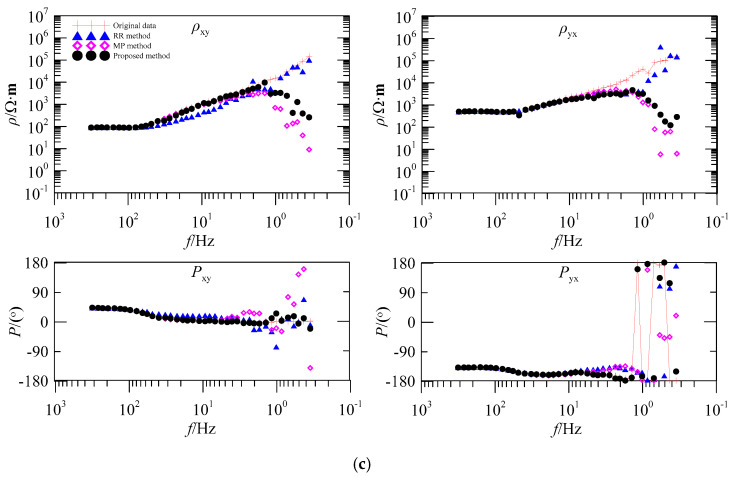
Comparison of apparent resistivity-phase curves of the measured sites (**a**) C41820, (**b**) EL22175, and (**c**) EL22211; red curve is for the original data, blue curve for the result obtained by the remote reference (RR) method, pink curve for the data filtered by the matching pursuit (MP) method, and the black curve for the result derived from the proposed method.

Figure 5 shows the comparison of the apparent resistivity-phase curves of the original data, remote reference (RR) method, matching pursuit (MP) method, and the proposed method. These measured sites (C41820, EL22175, and EL22211) analyses were subjected to the same type of interference (square, triangle, and pulse wave) in time domain waveforms. The sites were collected in the Luzong area, Anhui province, China. The collection time was 19 h for Figure 5a and 1 h for Figure 5b and c, respectively. The measured data were collected using a V5-2000 instrument (produced by Phoenix Company, Canada). As most geophysical information on these sites is concentrated in low-frequency bands, we only analyzed the low-frequency data in detail.

In Figure 5a, the apparent resistivity curves of the original data of $\rho_{xy}$ and $\rho_{yx}$ component appear to rise severely at 10 Hz–0.1 Hz. However, the curves dropping in the frequency band below 0.1 Hz, and the corresponding phase curve show severe mutation. Thus, the result belonged to the typical near-source effect, and its data reliability was completely unexplained. The sites on Figure 5b,c were conducted on the same line. The apparent resistivity curves of the original data in Figure 5b,c were about 45° asymptote rising, and the corresponding phase curves were all close to 0° and 180°. Given these sites were similar in their response when affected by strong interference, the results could not reflect the underground inherent electrical structure information.

Compared to the result obtained using the remote reference (RR) method, the apparent resistivity curve still kept rising, and the distortion data in the phase curve were not reduced. The result was because the RR method only eliminated noise by selecting between a reference site and the measured data distance. Under the influence of strong continuous interference, the RR method failed to produce the desired effect. Although the matching pursuit (MP) method could suppress large-scale strong interference in the time series, many low-frequency useful MT signals were filtered out. This caused an upward trend that did not ease, as in Figure 5a, and a downward trend in Figure 5b,c in the apparent resistivity curve in the low-frequency band. Meanwhile, the corresponding phase curve was more chaotic. Moreover, the result could not effectively reflect underground geoelectric information. According to the results, the proposed method can eliminate the section identified as MT interference and reconstruct a useful MT signal with high precision. The apparent resistivity-phase curve becomes stable and continuous, and its amplitude is also in the normal range. Thus, the result indicated that the denoised data was more reliable and reasonable.

### 3.6. Polarization Direction Analysis

**Figure 6:**
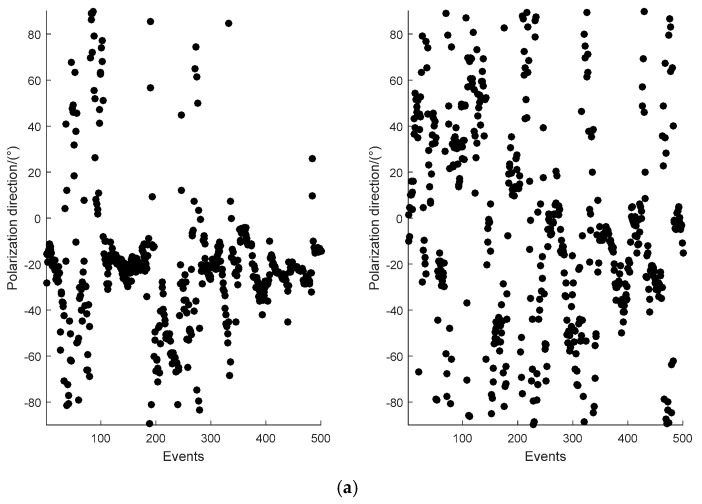

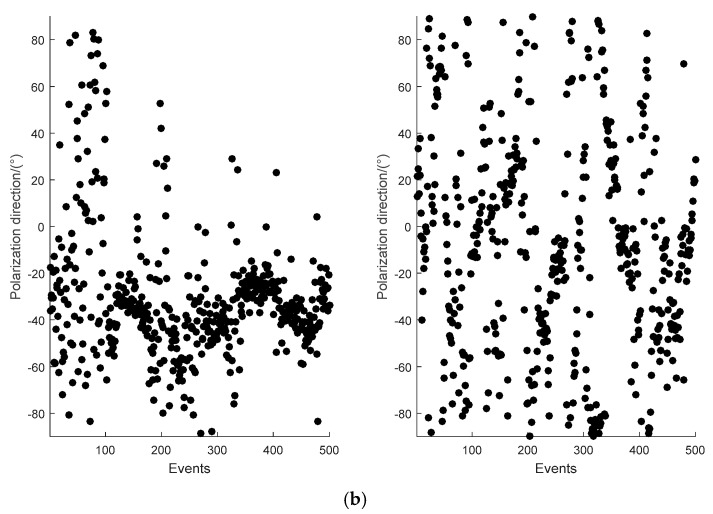
Comparison of the polarization direction at 0.3 Hz for site EL22211, (**a**) electric field data, and (**b**) magnetic field data.

Polarization direction [30] of the electromagnet field is an indicator for evaluating MT data quality. Thus, it was introduced to verify the effectiveness of the proposed method further. Figure 6 shows the comparison of electromagnetic polarization direction at a frequency of 0.3 Hz for the measured site EL22211 in the electric and magnetic fields, respectively. The left side of Figure 6a,b is the polarization direction of the original data, and the right side shows the polarization direction after it is denoised by the proposed method.

The polarization direction of the original data in the electric field and magnetic field was concentrated between −20° and −60° at 0.3 Hz, as shown in Figure 6. The active source area and strong interference waveforms were flooded with the original data. The polarization direction of the proposed method showed that electric and magnetic field data were relatively scattered and random, which was consistent with the polarization characteristics of natural field data. This result indicated that the proposed method was quite effective and the strong interference of the measured data had been suppressed.

## 4. Discussions

Geophysical methods are undoubtedly a powerful means to search for deep concealed mineral resources [31,32]. MT sounding is a significant method and has an irreplaceable role in the exploration of deep structures and metal deposits. However, weak MT data is highly susceptible to noise. For this reason, the development of methods to eliminate MT noisy signals and improve the quality of MT data will be beneficial to subsequent geoelectric structure analysis.

According to early literature on MT signal-noise separation, the MT data of time-frequency domain is analyzed and denoised [33,34]. These methods cannot distinguish between the signal and noise, and the useful data of low-frequencies are also filtered and blindly suppressed as noise, that is, the results cannot truly and reliably reflect the underground electrical structure. Further research in recent literature reveals that fractal, multifractal, entropy, and other ways of describing the characteristics of MT signal-noise are emerging. More robust characteristic parameters are used for the analyses, and good results have been obtained. However, this paper introduces only two characteristic parameters (IE-LZC), which are simple in an algorithm, fast in calculation, and easy to implement. The efficiency of MT signal-noise separation has improved with due consideration to data quality. For the mentioned measured site processing (Figure 5), the proposed method can be completed in a short time.

In this study, the IE and LZC are combined with a cluster method to distinguish the MT signal from interference. To validate the results, we first used sample library signal analysis to calculate the IE and LZC characteristic parameters of the known sample signals, and then we input the outcomes into FCM clustering (Figure 2). Considering the simple principle of the IE and LZC algorithm, these results supported the hypothesis that the IE-LZC and cluster methods were able to distinguish between the MT signal and interference (Figure 4). The MP algorithm is a signal sparse decomposition method, which primarily uses the linear operation of the atom vector to gradually approximate the signal vector, and it constantly iterates to achieve a given sparsity. The MP de-noising effect of two different atom types (Figure 1) indicates that the appropriate atom can be selected to match the corresponding signal for the mutation and stationary component of signals, where the mutation components are eliminated. Meanwhile, the useful signals are preserved.

We verified the Qinghai test site (Figure 3) and compared the results to ones achieved using the RR method and MP method for the measured sites (Figure 5). The proposed method could avoid over-processing and preserve more useful MT signals with improved MT data quality. However, the limitation of the paper was that when the signal and noise were gradually blurred, the clustering precision would drop sharply. Moreover, the number of iterations and atomic types need to be preset in the MP algorithm. If the intelligent algorithm is used to learn the MT data characteristics to improve the identification accuracy and adaptive selection of the parameters in the MP algorithm, it will significantly enhance the level of data processing.

## 5. Conclusions

A novel MT signal-noise separation method using information entropy (IE), Lempel-Ziv complexity (LZC), and matching pursuit (MP) algorithms was proposed. To remedy the low efficiency and to improve the accuracy of MT data processing in the existing techniques, the proposed method was applied to distinguish between interference and useful MT signals by analyzing the data from the Qinghai test site and other measured sites. The experimental results showed that the characteristic parameters and cluster method could effectively identify MT strong interference in time series sequence, while the MP de-noising could suppress results identified as MT strong interference while retaining the slow-change components of low-frequencies. At the same time, the data quality and apparent resistivity-phase curves were improved. The results obtained using the proposed method reflected the inherent electrical structure information more realistically, providing more reliable data for inversion.

## Figures and Tables

**Table 1:** MP de-noising performance of different atoms.

	NCC	SNR(dB)	MSE
sin/dct	0.9990	22.6268	0.0112
sym/db wavelet	0.9996	23.6209	0.0061
